# The effects of catchment and riparian forest quality on stream environmental conditions across a tropical rainforest and oil palm landscape in Malaysian Borneo

**DOI:** 10.1002/eco.1827

**Published:** 2017-03-21

**Authors:** Sarah H. Luke, Holly Barclay, Kawi Bidin, Vun Khen Chey, Robert M. Ewers, William A. Foster, Anand Nainar, Marion Pfeifer, Glen Reynolds, Edgar C. Turner, Rory P. D. Walsh, David C. Aldridge

**Affiliations:** ^1^ Department of ZoologyUniversity of CambridgeDowning StreetCambridgeCB2 3EJUK; ^2^ School of ScienceMonash UniversityJalan Lagoon Selatan47500Subang JayaSelangor Darul EhsanMalaysia; ^3^ Natural Disaster Research CentreUniversiti Malaysia SabahJalan UMS88400Kota KinabaluSabahMalaysia; ^4^ Forest Research Centre (Sepilok)Sabah Forestry DepartmentP.O. Box 140790715SandakanSabahMalaysia; ^5^ Department of Life SciencesImperial College London, Silwood Park CampusBuckhurst RoadAscotSL5 7PYUK; ^6^ Faculty of Science and Natural ResourcesUniversiti Malaysia SabahJalan UMS88400Kota KinabaluSabahMalaysia; ^7^ School of BiologyNewcastle UniversityRidley Building 2Newcastle upon TyneNE1 7RUUK; ^8^ The South East Asia Rainforest Research Partnership (SEARRP)Danum Valley Field CentreP.O. Box 6028291112Lahad DatuSabahMalaysia; ^9^ Department of GeographySwansea UniversitySwanseaSA2 8PPUK

**Keywords:** freshwater, habitat disturbance, oil palm, rainforest, riparian buffer, selective logging, Southeast Asia, water quality

## Abstract

Freshwaters provide valuable habitat and important ecosystem services but are threatened worldwide by habitat loss and degradation. In Southeast Asia, rainforest streams are particularly threatened by logging and conversion to oil palm, but we lack information on the impacts of this on freshwater environmental conditions, and the relative importance of catchment versus riparian‐scale disturbance. We studied 16 streams in Sabah, Borneo, including old‐growth forest, logged forest, and oil palm sites. We assessed forest quality in riparian zones and across the whole catchment and compared it with stream environmental conditions including water quality, structural complexity, and organic inputs. We found that streams with the highest riparian forest quality were nearly 4 °C cooler, over 20 cm deeper, had over 40% less sand, greater canopy cover, more stored leaf litter, and wider channels than oil palm streams with the lowest riparian forest quality. Other variables were significantly related to catchment‐scale forest quality, with streams in the highest quality forest catchments having 40% more bedrock and 20 times more dead wood, along with higher phosphorus, and lower nitrate‐N levels compared to streams with the lowest catchment‐scale forest quality. Although riparian buffer strips went some way to protecting waterways, they did not maintain fully forest‐like stream conditions. In addition, logged forest streams still showed signs of disturbance 10–15 years after selective logging. Our results suggest that maintenance and restoration of buffer strips can help to protect healthy freshwater ecosystems but logging practices and catchment‐scale forest management also need to be considered.

## 1 INTRODUCTION

Freshwater ecosystems are intricately linked with their surrounding terrestrial habitats. In the case of stream systems, all inputs of water, sediment, organic matter, and sunlight are strongly influenced by properties of the stream catchment and riparian zone, which in turn shape the structure, nutrient availability, and ecology of the stream habitat (Allan, 2004). Any changes in land use therefore have the potential to affect freshwater ecosystems fundamentally. Globally, it has been estimated that 65% of river habitats are under moderate to high threat from land‐use change (Vörösmarty et al., 2010). Freshwater ecosystems provide essential services for people, including water for drinking, homes, agriculture, and industry, as well as food resources such as fish and crustaceans. They also provide habitat for 6% of the world's species (Dudgeon et al., 2006), of which it is estimated that 10,000–20,000 are currently at risk of extinction (Strayer & Dudgeon, 2010; Vörösmarty et al., 2010). If the ecosystems and services provided by freshwaters are to be maintained and managed effectively, it is essential that the impacts of land‐use change and degradation on waterways are understood.

Southeast Asia, particularly the Sundaland region, which includes Borneo, has some of the highest rates of land‐use change in the world (Sodhi, Koh, Brook, & Ng, 2004) and had lost nearly 70% of its lowland forests by 2010 (Wilcove, Giam, Edwards, Fisher, & Koh, 2013). By 2009, just 25% of land in Sabah, Malaysian Borneo, was covered by intact forest, whilst 31% was degraded or severely degraded forest, much of which had been logged multiple times (Bryan et al., 2013). Increasingly, these logged forests are also being converted to timber, rubber, and, particularly, oil palm plantations (Wilcove et al., 2013). By 2010, 20% of Sabah's land area was being used to grow oil palm (Elaeis guineensis), and it is estimated that 62% of all plantations in Sabah have been established on land directly converted from forests (Gunarso, Hartoyo, Agus, & Killeen, 2013). Although selective logging only removes the largest trees of commercial species (mainly of the family Dipterocarpaceae), it is estimated that many more die, with 41% of remaining trees being uprooted and crushed and another 18% suffering damage to their crowns or bark (Pinard & Putz, 1996). In addition, bulldozers directly affect approximately 30–40% of any area being logged (Bryan et al., 2013). Skid trails, log landing areas, and logging roads, along with full‐scale conversion for agriculture, create large areas of exposed and compacted soil that are vulnerable to increased runoff and high rates of soil erosion (Brooks & Spencer, 1997; Douglas, 1999).

It is likely that logging and oil palm agriculture are having substantial impacts on freshwater systems in the region. A broad literature on the impacts of catchment‐scale and riparian land use exists for temperate freshwaters (e.g., reviewed by Allan, 2004; Tabacchi et al., 2000). But those impacts are less clear for tropical freshwater systems, which differ substantially from temperate ones in terms of rainfall and flooding regime, nutrient loads, biotic interactions, and normal levels of sediment and organic matter (Boulton et al., 2008; Dudgeon, 1999; Payne, 1986). In addition, the type and extent of land‐use changes being experienced in the tropics often differ from those in temperate regions. Temperate or tropical land‐use changes that result in larger areas of bare soil increase surface runoff, gully formation, and potential for flash floods and may cause permanently higher streamflow (Brooks & Spencer, 1997; Bruijnzeel, 2004; Douglas, 1999). This can increase sediment flow into streams, loss of nutrients from soils (Douglas, 1999; Malmer, 1996; Malmer & Grip, 1994) and streamwater nutrient, and mineral concentrations (Douglas, 1999). Loss of vegetation decreases water interception by canopy and leaf litter and reduces removal rates of water by transpiration, whilst soil disturbance and compaction reduces water infiltration (Bruijnzeel, 2004; Douglas, 1999). Loss or degradation of forest in the riparian zone may alter channel cross‐sectional size and shape, reduce inputs of woody debris, reduce shading and promote algal growth, change water chemistry, and remove the final barrier to sediment and nutrient inputs into streams (de Souza, Fonseca, Libório, & Tanaka, 2013; Dosskey et al., 2010; Fernandes, Souza, & Tanaka, 2013; Sweeney et al., 2004). It is uncertain how long it takes for freshwater ecosystems to recover from disturbance caused by land‐use change, with studies showing mixed results. Recovery to predisturbance sediment levels has been reported only 2 years after oil palm plantation establishment in Malaysia (DID, 1986, in Douglas, 1999). In contrast, studies in Kuala Lumpur (Lai 1992 and 1993, in Douglas et al., 1999) found that it took 8–20 years for erosion levels to return to normal, and streamflow had still not returned to normal 7 years after logging at another site in Peninsular Malaysia (Rahim & Zulkifli, 1994, in Bruijnzeel, 2004).

Several mitigation strategies have been proposed to reduce the impacts of land‐use change on freshwaters and aid recovery after disturbance. Reduced impact logging using practices such as stock mapping, skid trail planning, liana cutting, and avoiding slopes steeper than 25° (Pinard & Putz, 1996; Putz & Pinard, 1993; Putz, Sist, Fredericksen, & Dykstra, 2008) minimises damage to remaining forest and therefore nearby freshwaters compared to traditional mechanised approaches (Bruijnzeel, 2004; Chappell et al., 2008; Douglas, 1999; Walsh et al., 2011). Terracing of slopes, planting of cover crops, and appropriate road construction are also recommended for reducing erosion in oil palm plantations (RSPO, 2013). Retaining riparian vegetation and forest fragments in agricultural areas has been found to substantially reduce impacts on freshwater systems in a range of tropical regions (e.g., de Souza et al., 2013; Fernandes et al., 2013; Heartsill‐Scalley & Aide, 2003; Suga & Tanaka, 2012). Riparian buffer strips (protected zones of natural or non‐crop habitat left beside waterways) have been widely adopted as a mitigation strategy for reducing impacts of land‐use change on freshwaters, and they are one of the certification criteria for sustainable palm oil production under the Roundtable on Sustainable Palm Oil (RSPO, 2013). In Sabah, 20 m wide riparian buffers are required along all rivers measuring 3 m or more in width in order to maintain water volume and flow, prevent degradation of water quality, and damage to the aquatic environment (Sabah Water Resources Enactment, 1998) although these regulations are often poorly enforced and many rivers currently lack adequate, or indeed any, riparian buffers. In tropical ecosystems in particular, a consensus has not yet been reached on the most appropriate width for riparian buffers or the extent of forest cover across the wider catchment that needs to be retained in order to minimise limnological change. Furthermore, few studies have considered effects on a range of stream conditions simultaneously, or the effects of forest disturbance over multiple spatial scales (Allan, 2004). There have been calls for a greater consideration of potential changes to freshwaters in logged forest landscapes (Bruijnzeel, 2004), and research into the impacts of oil palm on freshwaters is very limited.

This study assesses how stream conditions, including sediment characteristics, water quality, channel structure, and organic inputs, change along a gradient of forest disturbance, comprising old growth forest, logged forest of varying quality, oil palm with riparian buffer strips of differing widths, and oil palm with no buffer strips in Sabah, Malaysian Borneo. We consider how stream environmental conditions vary in relation to quality of forest at the catchment scale and in the riparian zone, the effects of riparian buffer strips, and the rate at which streams recover after forest disturbance.

## 2 METHODS

### 2.1 Stream sites

**Figure 1:**
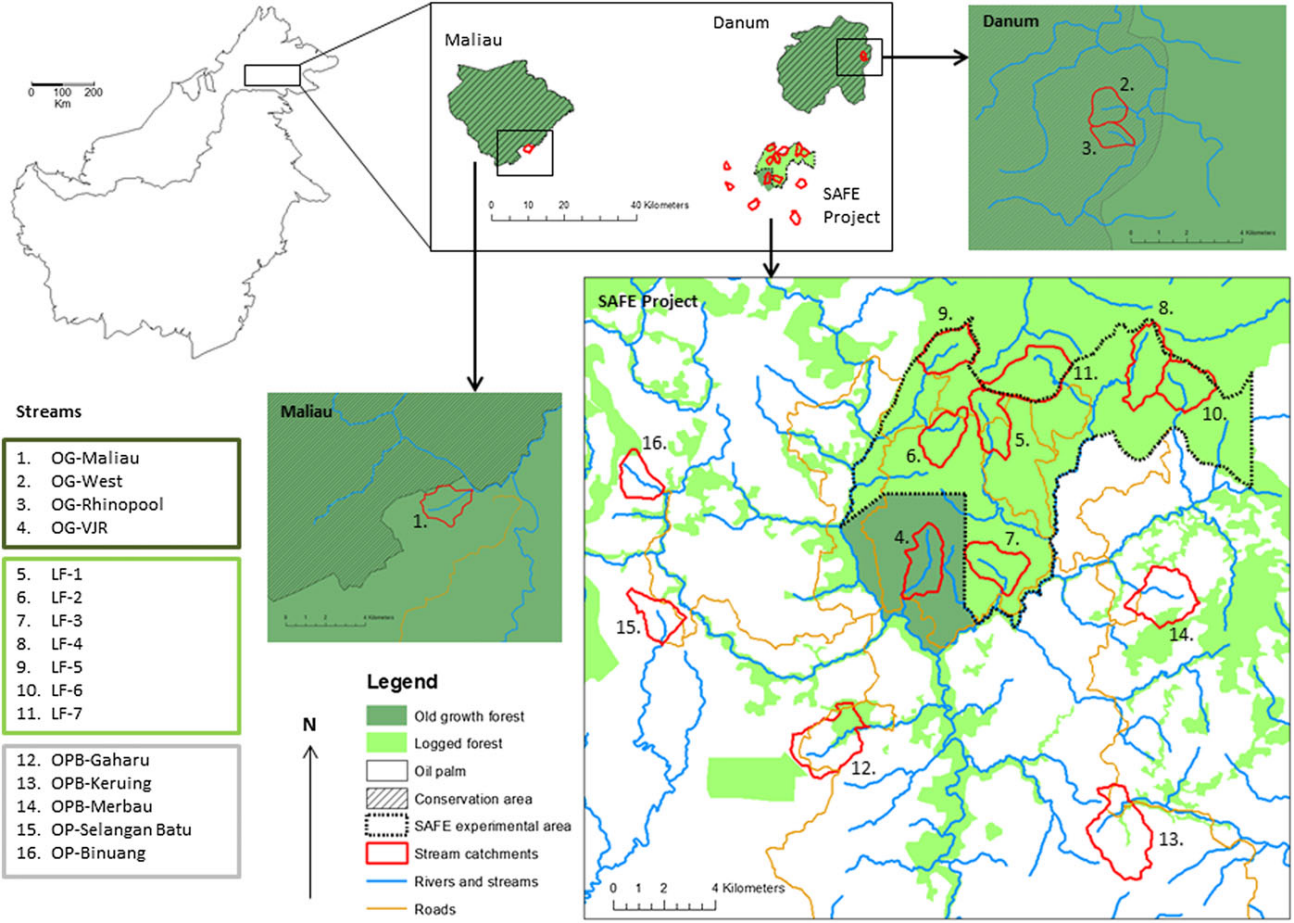
Schematic and map showing the location of the 16 stream sites used in our study within Sabah, Malaysian Borneo. The Borneo inset map was drawn using library “maps” in R statistical package (Becker & Wilks, 2015; R Core Team, 2014). All other maps were drawn using ArcMap 10.2.1 GIS software (Environmental Systems Research Institute [ESRI], 2014) using map layers developed from Landsat imagery (Ewers et al., 2011) and local maps and information from maps in Douglas et al. (1992) and Hansen et al. (2013). LF, logged forest; OG, old growth; OPB, oil palm with buffer; SAFE, Stability of Altered Forest Ecosystems

We conducted survey work in Sabah, Malaysian Borneo (Figure 1). The region has an equatorial climate with high annual rainfall and little seasonality, but with a tendency for drought from February to early May in major ENSO years (Walsh & Newbery, 1999). Mean annual rainfall at Danum Valley Field Centre 1985–2012 was 2,883 mm (Walsh et al., 2013), and 2,455 mm at the “Stability of Altered Forest Ecosystems” (SAFE) Project site near Tawau, 2012–2015 (Rory P.D. Walsh, unpublished data). The geology is similar across stream sites and comprises a mixture of sedimentary rocks including sandstones, mudstones, and tuff, and orthic acrisols are the dominant soil type (see Nainar, Bidin, Walsh, Robert, & Reynolds, 2015 for more information). The natural vegetation is lowland dipterocarp rainforest (Marsh & Greer, 1992).

We surveyed 16 streams (Figure 1) that were located at a mean altitude of 236 m asl ± SE 26 m and were matched according to slope (mean slope across the whole catchment of 18.24° ± SE 0.81°). In each stream, we started our survey work at matched points that had an upstream catchment size of 3.16 km^2^ ± SE 0.31 km^2^ and approximately 2 km of headwater flow. We will henceforth refer to these points as the “0‐m point” of each stream. Stream catchments were located across three research areas: the Danum Valley Conservation Area (117° 48.75′ E and 5° 01′ N), the Maliau Basin Conservation Area (116° 54′E and 4° 49′ N), and the SAFE Project site in an area of the Kalabakan Forest Reserve (116° 57′ E to 117° 42′ E and 4° 38′ N to 4°46′ N; Figure 1). The SAFE Project is a large‐scale, long‐term research project that is making use of government‐planned forest clearance and conversion to oil palm to investigate the impacts of land‐use change and forest fragmentation on ecosystems (see Ewers et al., 2011 for more information). We chose catchments in areas that had undergone different levels of habitat disturbance and conversion, which are typical of the major types of habitat change found in this region (Reynolds, Payne, Sinun, Mosigil, & Walsh, 2011).

1. Four streams in old‐growth lowland dipterocarp rainforest (old growth [OG]). Old‐growth forest sites were within the Danum Valley Conservation Area, the Maliau Basin Conservation Area, and a virgin jungle reserve (VJR) at the SAFE Project site (Figure 1). The two Danum Valley sites (OG‐West and OG‐Rhinopool) had never been logged. The Maliau Basin site (OG‐Maliau) had been very lightly logged (to provide timber for adjacent field centre buildings), and the VJR (OG‐VJR) had suffered minimal illegal felling (Ewers et al., 2011), but neither the VJR nor Maliau had experienced the extent of commercial selective logging characteristic of the wider region, and tree cover at the Maliau site remained similar to that at undisturbed sites (Hamzah Tangki, unpublished data from Maliau for PhD dissertation, University of Zurich, 2014).
2. Seven streams in forests that had been selectively logged to different extents (logged forest [LF]). Logged forest sites were located at the SAFE Project (Figure 1). At the time of the study, the “SAFE experimental area” was a continuous forest that had undergone a round of selective logging during the 1970s that removed approximately 113 m^3^ of hardwood timber per hectare and multiple rounds from the late 1990s–2000s that removed a further 66 m^3^ ha^−1^ (LF‐1, LF‐2, LF‐3, LF‐4, LF‐5, and LF‐6), although in the case of LF‐7, this second round was only a single harvest of 37 m^3^ ha^−1^ (Fisher et al., 2011; Pfeifer et al., 2015; Struebig et al., 2013). Although logging had been completed at the same time across the landscape, the logged forest sites were very heterogeneous with patches of forest with closed canopy interspersed with early regrowth, gaps, and roads.
3. Three streams in oil palm plantations with forested riparian buffer strips remaining beside the streams (oil palm with buffer [OPB]). Oil palm sites were located in areas of mature oil palm (planted between 1999 and 2009) near the SAFE Project experimental area (Figure 1). Oil palms are usually planted 9 m apart, with a cover crop (often leguminous) grown between to help decrease soil erosion and nutrient loss (Corley & Tinker, 2003). The palms had not yet grown sufficiently to give a closed canopy (Luskin & Potts, 2011). All oil palm stream catchments were predominantly planted with oil palm but varied in the amount of forest cover and riparian buffer strip remaining in the catchment. The OPB‐Gaharu had a wide riparian buffer strip (mean ~ 331 m, minimum ~75 m) on each side of the stream. The OPB‐Keruing had a medium‐width riparian buffer strip (mean ~ 68 m, minimum ~33 m) on each side of the stream. The OPB‐Merbau had a narrow riparian buffer strip (mean ~ 26 m, minimum ~2 m) on each side of the stream.
4. Two streams (OP‐Binuang and OP‐Selangan Batu) in oil palm plantations with no buffer strips (oil palm no buffer [OP]). These were located in the same regions as detailed (in 3) above.

We sampled more streams in logged forest than in old‐growth forest and oil palm because logged forest sites were expected to show greater habitat heterogeneity, and it was important to ensure that the sites chosen covered a range of forest qualities. Forest quality varies continuously within our broad habitat categories (old‐growth forest [OG], logged forest [LF], oil palm with buffers [OPB], and oil palm no buffers [OP]), and some categories encompass more variation than others. We therefore conducted analyses using continuous measures of forest quality rather than these simplified categories.

Sites were surveyed before forest clearance, and conversion to oil palm occurred at the SAFE Project, and so they therefore form a valuable baseline data set for later comparison with postconversion data. Table S1 gives details of how each stream will be affected by proposed future logging at the SAFE Project.

### 2.2 Forest quality

We assessed riparian forest quality in each of the 16 streams at 50‐m intervals, for 500‐m upstream of the “0‐m point.” At each survey point, measurements were taken 10 m into the forest or oil palm on both sides of the stream. Canopy openness was measured using a spherical densiometer (with measurements directed upstream, downstream, towards, and away from the stream, and then averaged; Lemmon, 1956). Tree density was measured using a hand‐held relascope (Bitterlich, 1984), which is based on the angle‐count sampling method. To allow for lower tree numbers where the stream flowed, trees were counted in a 180° turn from upstream to away from the stream to downstream; the resulting count was then doubled to represent a full turn. Values were converted to an estimate of basal area (m^2^ ha^−1^) by doubling the value again. Forest quality and percentage cover of vines in the canopy within 10 m around the survey point were assessed visually. Forest quality was scored using the SAFE Project forest quality scale: 0 = oil palm; 1 = very poor—no trees, open canopy with ginger or vines, or low scrub; 2 = poor—open with occasional small trees over ginger or vine layer; 3 = OK—small trees fairly abundant or canopy at least partially closed; 4 = good—lots of trees, some large, and canopy closed; 5 = very good—closed canopy with large trees, no evidence of logging (Ewers et al., 2011; Pfeifer et al., 2015). Measurements were made once at each site in June–December 2011–2013, and repeated at all sites except OG‐West and OG‐Rhinopool in May–August 2014. Measurements were averaged to give a single value of each variable for each stream.

To quantify forest quality across the whole stream catchment, we used forest stand structure maps developed by Pfeifer et al. (2016). Maps showed mean above‐ground living biomass (AGB [t/ha]), leaf area index (LAI, defined as leaf area per ground area), and percentage forest cover (FCover) values within a 25‐m^2^ pixel. They were produced by modelling the relationship between on‐the‐ground measurements from forest quality plots (*n* = 193, taken in 2010 and 2011) and the corresponding spectral intensity, spectral vegetation indices and texture data from RapidEye™ satellite images (taken during 2012 and 2013), and upscaling the relationship for each pixel across the extent of the study area (for full details, please refer to Pfeifer et al., 2016). To assess forest quality within each stream catchment, we first calculated catchment areas using an ArcMap Hydrology toolbox (Environmental Systems Research Institute [ESRI], 2014) with an ASTER Digital Elevation Model (ASTER GDEM is a product of METI and NASA), and the start point of the catchment (snapping point) set to the 0‐m point in our catchments. These methods use topography information to calculate the likely path of flow and accumulation of water over the landscape and therefore delineate streams and catchments. Once catchment areas had been mapped, we used the library “raster” (Hijmans, 2014) in R statistical software (R Core Team, 2014) to clip the AGB, LAI, and FCover maps to each of the catchment areas and compute mean forest quality values (meanAGB, meanLAI, and meanFCover) for each one. In the case of OG‐West and OG‐Rhinopool, the stream catchments were obscured by cloud, making it impossible to calculate forest quality values for these catchments. Instead, we used forest quality values for the entire Danum Valley Conservation Area. As the Danum Valley area is a continuous forest that has never been logged or disturbed, it is very homogenous in cover and structure and is likely to offer a good approximation for the OG‐West and OG‐Rhinopool catchments.

### 2.3 Stream environmental variables

Measurements of a wide range of stream environmental variables were made once at each stream in April–August 2012, November–December 2012, or April–June 2013, in nonflood conditions along 200‐m transects starting at the 0‐m point and going upstream. Water chemistry variables, including temperature, pH, conductivity (Hanna Combo pH and EC Meter), and dissolved oxygen (Hach‐Lange HQ40 digital DO meter), were measured at five points in each stream (0, 50, 100, 150, and 200 m upstream of the 0‐m point). Stream structural variables including canopy openness over the stream, wetted width, total channel width, maximum depth, maximum velocity, sediment cover, and leaf litter were measured every 10 m. Canopy openness was measured from the middle of the stream in four directions (upstream, downstream, left, and right at each point) using a spherical densiometer. Channel width and wetted width of the stream were measured using a tape measure, and maximum depth was measured using a ruler. We measured maximum velocity at the fastest flowing part of the stream at each measurement point using a 2‐m string, tennis ball, and stopwatch. The time taken for the ball to travel 2 m was recorded three times and then averaged. We assessed sediment size in a 50‐cm‐wide band across the wetted width of the stream using percentage cover within five size categories: bedrock, large rocks (heavy, need two hands to move), small rocks (could pick up in one hand), pebbles, and sand. To assess the amount of leaf material retained within the stream (e.g., caught between rocks), we collected leaves from a 20‐cm‐wide band across the wetted width of the stream at each 10‐m point. Leaves were oven‐dried to a constant weight, which was then recorded.

In addition to point measurements every 10 m, we characterised the entire stream channel section between successive 10‐m points in terms of percentage cover of dead tree trunks (henceforth shortened to dead wood), rapids, riffles, and pools. If water was still or near‐still with no ripples, we defined the area as a pool; if water was moving and the surface was rippled, it was defined as a riffle; if water was moving fast enough to give white water, we defined it as a rapid. For analysis, we calculated the percentage contributions of rapids, riffles, and pools to this water total.

Water samples (~500 ml in a plastic bottle) were taken at the 0‐m point during nonflood conditions. Samples were taken approximately monthly for a subset of streams (LF‐1, LF‐2, LF‐3, LF‐4, LF‐5, LF‐6, LF‐7, OG‐VJR, and OP‐Selangan Batu) between 2011 and 2014 (giving between 13 and 29 samples from each stream) and on a single occasion for another subset of streams (OP‐Gaharu, OP‐Keruing, OP‐Merbau, and OG‐West) in 2014 (giving one sample for each stream). Samples were kept frozen and later analysed for nitrate‐N and phosphorus content using HACH nitrate and phosphate pocket colorimeters. We analysed Nitrate‐N using the cadmium reduction method (APHA, 2005), and reactive‐P was analysed using the acidic molybdenum‐blue method (APHA, 2005). Unfiltered samples were used unless they were very turbid.

### 2.4 Statistical methods

All statistical analyses were conducted using the R statistical package (R Core Team, 2014). As forest quality variables were nonindependent, we used principal component analysis (PCA) on the mean values for each variable to summarise the major axes of variation in forest quality. We ran separate PCAs for the riparian and catchment forest quality variables to produce summary riparian and catchment forest quality variables. Riparian PC1 and Catchment PC1 were used as forest quality variables in subsequent analyses.

We used linear mixed effects models (library “lme4,” Bates, Mächler, Bolker, & Walker, 2015) with random intercepts to assess individual relationships between riparian and catchment forest quality and each instream environmental variable measured. In each model, we treated the specific instream environmental condition as the response variable and either riparian (Riparian PC1) or catchment forest quality (Catchment PC1) as the fixed effect and stream identity as a random effect to take account of nonindependence of multiple measurements within a stream. Model residuals were checked for homoscedasticity and normality, and transformations were used where necessary to ensure that model assumptions were met. All percentage cover data were normalised using an arcsine square root transformation prior to analysis. For full details of statistical tests, refer to Table 2. We used log‐likelihood ratio tests to generate *p* values to assess model significance. Original data, fitted models, and 95% confidence intervals (CI) of the model were plotted using library “ggplot2” (Wickham, 2009, with reference to Chang, 2013).

## 3 RESULTS

### 3.1 Riparian and catchment forest quality

Principal component analysis produced summary scores for catchment and riparian forest quality. In the riparian PCA, the first principal component (Riparian PC1) explained 77.6% of the variation in measurements of riparian forest quality. Riparian PC1 scores were multiplied by −1 to make the scale more readily interpretable from low to high forest quality. In the catchment PCA, the first principal component explained 92.1% of the variation in AGB, LAI, and FCover measurements across catchments. Loadings of each of the original forest quality measurements on each principal component are shown in Table S2. Catchment PC1 and Riparian PC1 scores were correlated (Pearson's *r* = .57, *t* = 2.60, *df* = 14, *p* = .0211). Despite this, there were substantial differences in Riparian PC1 and Catchment PC1 scores for some streams, particularly the oil palm and oil palm buffer streams (Figure S1), indicating that the riparian and catchment scales should be considered separately in analyses.

### 3.2 Responses of stream variables to riparian forest quality

**Figure 2:**
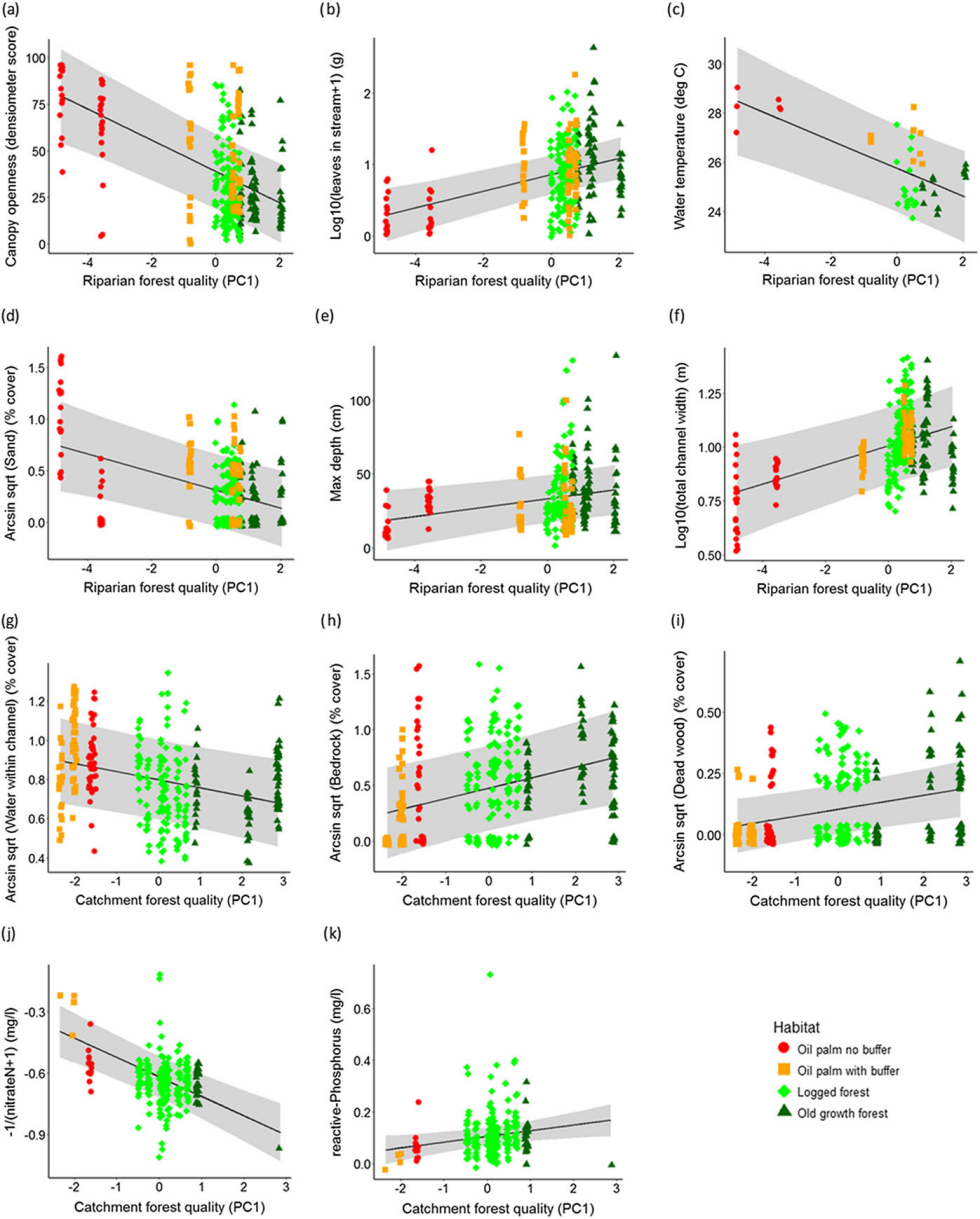
Relationship between (a–f) riparian forest quality PC1 and (g–k) catchment forest quality PC1 and stream environmental conditions. Points (jittered to aid viewing and coloured according to habitat type) show original repeat measures within each stream, whilst lines and 95% confidence intervals show results of mixed effects models (see Table 2)

Streams with high riparian forest quality (high Riparian PC1 scores) had significantly higher canopy cover at the centre of the stream, more leaf litter found lodged within the stream, and lower water temperatures than streams that had lower riparian forest quality (Figure 2a–c, Table 2). The model results suggest that streams with the highest riparian forest quality had over 10 times as much trapped instream leaf matter and were nearly 4 °C cooler than oil palm streams with the lowest riparian forest quality (Figure 2b and 2c). Water temperature and canopy openness also decreased with rising catchment forest quality (Catchment PC1), but the effect was less significant (Table 2). In terms of differences between broad habitat types (OG, LF, OPB, and OP), the results suggest that logged forest and old‐growth streams were similar in water temperature and instream canopy openness, whereas oil palm buffer streams were warmer, with a more open canopy, but less so than the nonbuffered oil palm streams (Figure 2a–c, Table 1). Oil palm buffer streams and logged forest streams were the most similar in their stocks of submerged leaves, although oil palm streams without buffers had substantially fewer leaves and old‐growth forest streams had substantially more (Table 1).

**Table 1:** Riparian forest quality, catchment forest quality, and stream environmental variables for the 16 streams used in this study. Values show the mean ± standard deviation for all the streams within each of four broad habitat categories: oil palm no buffer (OP), oil palm with buffer strips (OPB), logged forest (LF), and old‐growth forest (OG)

		Streams			
		Low forest quality	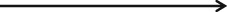		High forest quality
		OP (*n* = 2)	OPB (*n* = 3)	LF (*n* = 7)	OG (*n* = 4)
Riparian forest quality	SAFE forest quality scale (score 0–5)	0.03 ± 0.05	2.47 ± 0.47	2.72 ± 0.27	3.35 ± 0.62
	Vines (% cover)	0.34 ± 0.48	46.05 ± 2.20	45.71 ± 8.74	38.00 ± 15.53
	Tree basal area (m^2^/ha)	0	18.26 ± 7.39	17.66 ± 3.46	28.00 ± 8.70
	Canopy openness (score 0–96)	51.07 ± 25.1	16.74 ± 6.80	12.44 ± 3.34	7.77 ± 2.38
	**Riparian PC1**	−4.20 ± 0.90	0.15 ± 0.84	0.39 ± 0.24	1.30 ± 0.54
Catchment forest quality	Above ground biomass (AGB; t/ha)	2.49 ± 0.58	1.47 ± 0.34	5.16 ± 0.70	18.68 ± 6.30
	Leaf area index (LAI)	2.44 ± 0.05	2.22 ± 0.19	3.74 ± 0.32	4.50 ± 0.29
	Forest cover (%)	55.11 ± 0.25	48.47 ± 1.75	69.00 ± 2.81	81.26 ± 5.98
	**Catchment PC1**	−1.58 ± 0.06	−2.12 ± 0.19	0.11 ± 0.39	2.19 ± 0.92
Stream environmental conditions	Water temperature (°C)	28.22 ± 0.07	26.86 ± 0.36	25.02 ± 0.93 (*n* = 6)	24.99 ± 0.67
	Dissolved oxygen (mg/L)	8.10 ± 0.09	7.98 (*n* = 1)	8.06 ± 0.25 (*n* = 5)	8.04 (*n* = 1)
	pH	7.75 ± 0.16	7.89 ± 0.34	8.15 ± 0.28 (*n* = 6)	7.87 ± 0.46
	Conductivity (μS)	90.00 ± 7.07	58.67 ± 17.89	118.19 ± 64.94 (*n* = 6)	113.86 ± 38.29
	Nitrate‐N (mg/L)	0.74 (*n* = 1, with multiple measures per site)	2.69 ± 2.63 (*n* = 3, with a single measure per site)	0.64 ± 0.63 (*n* = 7, with multiple measures per site)	0.56 ± 0.31 (*n* = 2, one with multiple measures per site and one with a single measure)
	Reactive‐P (mg/L)	0.059 (*n* = 1, with multiple measures per site)	0.010 ± 0.0068 (*n* = 3, a single measure per site)	0.108 ± 0.108 (*n* = 7, with multiple measures per site)	0.098 ± 0.05 (*n* = 2, one with multiple measures per site and one with a single measure)
	Time taken for ball to move 2 m (s)	5.35 ± 1.79	6.65 ± 0.41	4.33 ± 3.28	9.76 ± 7.87
	Channel width (m)	6.77 ± 1.15	10.50 ± 1.64	12.18 ± 2.95	10.88 ± 3.43
	Wetted width (m)	4.18 ± 1.32	5.93 ± 0.23	5.61 ± 1.54	5.85 ± 1.86
	Maximum depth (cm)	23.24 ± 14.19	28.16 ± 7.14	33.99 ± 8.19	41.01 ± 12.37
	Submerged leaves dry weight (g)	1.73 ± 0.26	14.6 ± 6.67	12.48 ± 6.19	22.48 ± 20.43
	Instream canopy openness (score 0–96)	73.43 ± 15.31	49.60 ± 8.87	30.39 ± 13.04	28.20 ± 5.72
	Water within channel (% cover)	60.17 ± 4.49	63.83 ± 15.79	45.75 ± 8.91	46.00 ± 8.78
	Rapids (% cover)	1.96 ± 2.77	0.36 ± 0.32	17.52 ± 13.16	6.09 ± 5.38
	Riffles (% cover)	42.29 ± 3.66	47.25 ± 20.85	46.06 ± 18.45	27.72 ± 10.92
	Pools (% cover)	55.75 ± 6.43	52.39 ± 21.16	36.43 ± 2.80	66.19 ± 10.96
	Dead wood (% cover)	2.75 ± 3.89	0.33 ± 0.38	3.5 ± 1.55	4.94 ± 3.38
	Bedrock (% cover)	24.88 ± 36.06	7.90 ± 7.14	33.16 ± 11.51	37.50 ± 12.90
	Large rocks (% cover)	6.22 ± 8.67	6.21 ± 4.90	8.16 ± 4.03	13.93 ± 7.10
	Small rocks (% cover)	1146 ± 6.95	23.39 ± 11.61	18.23 ± 8.41	15.24 ± 2.30
	Pebbles (% cover)	16.83 ± 1.49	39.52 ± 22.51	29.86 ± 7.45	24.29 ± 13.71
	Sand (% cover)	40.37 ± 49.86	22.98 ± 6.35	10.69 ± 3.49	9.05 ± 2.97

*Note*. Riparian PC1 and Catchment PC1 values are calculated from principal component analysis (PCA) of riparian and catchment forest quality variables, respectively (see Section 2.4 for more details). The designation of streams into the four habitat types is shown in Figure 1. The number of streams (*n*) used to calculate each value is the number shown in parentheses in the heading, unless otherwise stated within the body of the table. Multiple values were taken in each stream (as described in methods), unless specifically listed as single measurements in the body of the table.

*Note*. SAFE, Stability of Altered Forest Ecosystems.

Streams with high riparian forest quality also had lower percentage cover of sand on the stream bed and a greater maximum depth and total channel width than streams with lower riparian forest quality (Figures 2d–f, Table 2). High‐quality forest streams had approximately 2% sand on the stream bed, compared to a modelled result of 45% in the lowest quality oil palm streams, although there was substantial variation between streams. Across broad habitat types, only logged forest streams were similar to old‐growth forest streams in terms of sand cover. Sand cover was higher in the oil palm streams, even including those which had riparian buffers (Figure 2d, Table 1). Streams with the highest quality riparian vegetation had a maximum depth over 20 cm deeper than that modelled for streams with the lowest riparian forest quality. These results correspond to a progressively increasing maximum depth across the habitat types from oil palm through to old‐growth forest (Figure 2e, Table 1). Model results suggest that the highest forest quality streams were double the total width, from bank to bank, than the lowest forest quality streams, but that there was no significant difference in wetted width between high and low quality streams (Figure 2f, Table 2). This means that there were more dry areas in the channel of higher forest quality streams and that percentage cover of the channel by water was significantly related to forest quality. Oil palm streams without riparian buffers were only just over half as wide as streams in old growth forest, logged forest, and oil palm streams with buffers, all of which had similar channel widths. However, although old‐growth forest and logged forest streams had similar percentage cover of water within the channel, oil palm streams had higher percentage water cover (fewer dry areas) than the forested streams and this difference was found in oil palm streams with and without riparian buffers (Figures 2g, Table 1).

**Table 2:** Model equation and details of variables (including transformations) used in mixed effects models, along with results of log‐likelihood ratio test comparisons of mixed model results with null models to assess significance of relationships between catchment and riparian forest quality and stream environmental variables

For mixed effects models of the form: lmer (transformed response variable ~ forest quality explanatory variable + [1|Stream])			
Transformed response variable	Forest quality explanatory variable	Results of log‐likelihood ratio test	
		χ^2^	*p*
Water temperature	Catchment PC1	9.3494	.0022^b^
	Riparian PC1	11.183	.0008^c^
Dissolved oxygen	Catchment PC1	0.3929	.5308
	Riparian PC1	0.0038	.9595
pH	Catchment PC1	0.0002	.9885
	Riparian PC1	0.3929	.5308
Conductivity	Catchment PC1	2.4513	.1174
	Riparian PC1	0.1647	.6849
−1/(Nitrate‐N + 1)	Catchment PC1	22.188	<.0001^c^
	Riparian PC1	0.9095	.3402
Reactive‐P	Catchment PC1	5.0749	.0243^a^
	Riparian PC1	1.789	.1811
−1/(flow time)^0.5 (time for a ball to move 2 m)	Catchment PC1	0.413	.5205
	Riparian PC1	0.0005	.9823
Log10 (total channel width)	Catchment PC1	0.7631	.3824
	Riparian PC1	8.3182	.0039^b^
Log10 (wetted width)	Catchment PC1	0.0004	.9845
	Riparian PC1	2.7478	.0974
Maximum depth	Catchment PC1	3.5281	.0603
	Riparian PC1	4.5558	.0328^a^
Log10 (submerged leaves weight + 1)	Catchment PC1	3.6944	.0546
	Riparian PC1	13.424	.0002^c^
Instream canopy openness	Catchment PC1	8.9976	.0027^b^
	Riparian PC1	16.176	<.0001^c^
Arcsin square root (% cover water stream channel)	Catchment PC1	5.1588	.0231^a^
	Riparian PC1	3.1049	.0781
Arcsin square root (% cover of rapids)	Catchment PC1	0.6447	.4220
	Riparian PC1	0.9061	.3411
Arcsin square root (% cover of riffles)	Catchment PC1	2.7009	.1003
	Riparian PC1	0.9683	.3251
Arcsin square root (% cover of pools)	Catchment PC1	0.8109	.3679
	Riparian PC1	0.0787	.7790
Arcsin square root (% cover of dead wood)	Catchment PC1	7.7975	.0052^b^
	Riparian PC1	0.3899	.5323
Arcsin square root (% cover of bedrock)	Catchment PC1	7.1287	.0076^b^
	Riparian PC1	1.4152	.2342
Arcsin square root (% cover of large rocks)	Catchment PC1	3.1435	.0762
	Riparian PC1	2.2871	.1305
Arcsin square root (% cover of small rocks)	Catchment PC1	0.682	.4089
	Riparian PC1	1.7454	.1865
Arcsin square root (% cover of pebbles)	Catchment PC1	0.8835	.3473
	Riparian PC1	1.1906	.2752
Arcsin square root (% cover of sand)	Catchment PC1	4.0783	.0434^a^
	Riparian PC1	8.1223	.0044^b^

*Note*. *n* = 16 streams (unless stated otherwise in Table 1), with multiple repeat measures in each stream (see Section 2).

Significant results are denoted by the following:

a *p* < .05,

b *p* < .01, and

c *p* < .001.

### 3.3 Responses of stream variables to catchment forest quality

Other instream environmental variables showed significant relationships with catchment‐scale forest quality rather than riparian forest quality. Modelled results indicate that streams with the highest catchment forest quality had 46% bedrock cover compared to only 6% in the lowest quality catchment streams, and had over 20 times more dead wood than lowest forest quality oil palm streams (Figures 2h–i). Considering broad differences between major habitat types, logged forest, and old growth forest streams had similarly high levels of exposed bedrock, with lower levels in oil palm streams. Levels of dead wood in the streams declined steadily from old‐growth through to logged forest, then oil palm, with the lowest levels in the oil palm streams with buffers (Table 1).

Nitrate‐N levels were significantly lower in higher quality catchment streams with models suggesting that nitrate values were about 12 times lower in the highest quality streams than in the lowest quality oil palm streams (Figure 2j, Table 2). Phosphorus showed the opposite trend, with levels being three times higher in streams with high catchment forest quality scores than those in the lowest quality oil palm catchment (Figure 2k, Table 2), although the difference was only approximately 0.1 mg/L. Across the broad habitat types, logged and old‐growth forests appear most similar in terms of nitrate‐N and phosphorus levels, with oil palm streams showing higher nitrate‐N and lower phosphorus levels, with the highest values recorded at oil palm sites with riparian buffers (Table 1). All nitrate and phosphorus values, however, are well below pollution threshold levels.

We found no significant differences in other aspects of water quality (dissolved oxygen, pH, and conductivity) in relation to either riparian or catchment quality, nor in a range of other water flow and sediment conditions (velocity; wetted width; percentage cover of rapids or pools; percentage cover of large rocks, small rocks, or pebbles; Table 2).

## 4 DISCUSSION

Our study is the first to demonstrate how riparian and catchment forest quality affect stream environmental variables across a habitat degradation landscape in Southeast Asia. We show that forest quality at both the riparian and catchment scales are significantly related to stream environmental conditions and that different conditions are affected by habitat quality across different scales. In accordance with other studies in the region, our results indicate that the impacts of selective logging are still evident in stream environmental conditions over 10 years after logging, because logged forest streams showed differences in conditions to the old‐growth forest streams. In turn, oil palm streams with riparian buffers retained more natural stream conditions than oil palm streams without buffers, illustrating the importance of retaining or restoring riparian buffers for freshwater management in these systems. However, they still differed from forested streams in many of their channel characteristics and some of their chemical conditions, suggesting that riparian buffer strips alone are not sufficient to protect streams fully from the impacts of oil palm agriculture.

Specifically, we found that streams with higher quality riparian forest had significantly lower canopy openness over the stream, lower water temperatures, and higher levels of leaf material in the water. They also had lower percentage cover of sand, greater maximum depth, and greater channel widths compared to streams with lower quality riparian habitat. Other variables showed stronger trends with forest quality across the catchment‐scale. Percentage cover of bedrock, dead wood, and phosphorus levels were significantly higher in streams with higher catchment forest quality, although water cover within the stream channel and nitrate‐N levels were lower.

### 4.1 Responses of stream variables to riparian forest quality

Loss of tree cover in the riparian zone through selective logging or complete clearance for oil palm reduces the canopy cover above the stream, leading to higher canopy openness scores over the centre of the stream, and lower availability of leaves to fall into the water. As well as increasing light levels and reducing leaf input, lower riparian forest cover reduces shading and consequently results in higher water temperatures (e.g., Kiffney, Richardson, & Bull, 2003; Moore, Spittlehouse, & Story, 2005). In our study, light exposure almost doubled and water temperature was approximately 4 °C higher in streams with lowest riparian forest quality compared to those of the highest quality. Water temperature was also significantly correlated with catchment‐scale forest quality, and other studies have found that upstream forest cover, for at least a few hundred metres, is important for stabilising downstream water temperatures (Scarsbrook & Halliday, 1999; Storey & Cowley, 1997). This may be because temperature of runoff water into streams is affected by temperatures across the catchment. Air temperatures have been found to increase by up to 6.5 °C when forest is converted to oil palm (Hardwick et al., 2015) and with the average surface temperature in Borneo predicted to increase by up to 3–4 °C by 2081–2100 relative to 1986–2005, as a result of climate change (Intergovernmental Panel on Climate Change [IPCC], 2014), higher water temperatures are likely to become increasingly common.

Streams with lower riparian forest quality had narrower channels, lower maximum depths, and higher percentage cover of sand on the streambed. Narrow channels were a feature of the oil palm streams without buffer strips, perhaps because reduced riparian shading may allow increased growth of understory vegetation on the stream edge, which hold the banks together and reduce erosion (Sweeney et al., 2004). The maximum depth of oil palm streams was almost half that of streams in old‐growth forest. Despite allowing growth of bank‐stabilising plants near the stream edge, reduced vegetation cover in the wider riparian landscape increases the likelihood of there being areas of bare ground from which soil can be eroded, and fewer leaves, roots, and less leaf litter to act as a barrier to its transport directly into the stream (Bruijnzeel, 2004). It is well established that increased terrestrial disturbance can lead to increased sediment levels in streams. Sediment loadings up to 50 times higher than normal levels have been recorded in disturbed sites in Malaysia (Douglas, Greer, Bidin, & Spilsbury, 1993), although high sediment yields were found in streams draining both newly planted and mature (>10 years old) oil palm plantations in Indonesia (Carlson et al., 2014). In addition, clear‐felling forest and replacing it with cocoa and oil palm increased sediment loads by nearly 15 times (from a mean of 28 t/km^2^ to 414 t/km^2^ in one of the streams; DID, 1986; DID, 1989, in Douglas, 1999) relative to prelogging conditions. Such increases in stream sediment loads probably contributes to high levels of sand and silt settlement on the streambed, resulting in a shallower average depth, infilling of the deepest pools and overall simplification of the stream bed habitat (Allan, 2004).

### 4.2 Responses of stream variables to catchment forest quality

Several stream variables showed significant relationships with catchment‐scale forest quality rather than riparian forest quality. Percentage cover of bedrock, dead wood, and levels of phosphorus in the water were significantly higher in streams with higher catchment forest quality, although levels of nitrate‐N were lower. Nitrates are readily leached from tropical soils (Payne, 1986), particularly when land is disturbed by clearance (Malmer & Grip, 1994), and so levels in stream water may be high until vegetation regrowth removes more nitrogen from soil water (Malmer & Grip, 1994). The elevated nitrate levels in oil palm streams most likely resulted from runoff of fertilisers that are added to oil palm plantations (Yusoff & Hansen, 2007). However, local guidelines encourage the use of recycled biomass (e.g. cut fronds, empty fruits bunches, cover crops) and minimal use of inorganic fertilisers (Malaysian Palm Oil Board 2014), and fertiliser application at our sites appeared to be targeted through use of slow‐release fertilisers from semipermeable bags (personal observation). It is also noteworthy that although we detected significant differences between sites, nitrate levels were low. Levels were generally lower than those found in a study of oil palm and forested control streams in Sarawak (mean nitrate‐N in oil palm 2.70 mg/L, cf., 1.71 mg/L in our study, and mean nitrate in forest of 1.92 mg/L, cf., 0.60 mg/L in our study, Mercer, Mercer, & Sayok, 2013), and (apart from one outlier) our results are still within recommended limits for sensitive aquatic species on the basis of Malaysian National Water Quality Standards (Ministry of Natural Resources and Environment Malaysia, 2014). They are also substantially lower than values recorded in agricultural catchments in Eastern England over recent decades, which have often exceeded the maximum 50 mg/L level required for drinking water (Skinner et al., 1997).

Phosphorus levels showed the opposite trend to nitrate‐N levels, with highest phosphorus values in the logged and old‐growth forest sites and lower levels in oil palm, despite fertilisers being added to plantations. This may be because phosphorus is needed in large quantities by rapidly growing plants (de Souza et al., 2013; Dosskey et al., 2010), which would include oil palm, scrub, and forest regrowth in the low‐quality forest streams. However, less is taken up by slow‐growing, mature vegetation, perhaps resulting in the higher levels observed in the old‐growth and less disturbed logged forest sites. In addition, high‐throughflow and runoff rates in more disturbed catchments (Bruijnzeel, 2004; Douglas, 1999) may dilute the phosphorus released from weathering of underlying rocks and organic matter breakdown. However, the numerical difference was small and therefore unlikely to have substantial impacts on the stream system. Inputs of tree trunks into streams depend entirely on supply of dead trees from the surrounding forest and, because wood is often carried a long way downstream, particularly in flood events, it makes sense that higher levels of forest at the catchment‐scale gave higher levels of wood in both our study and in others (Cadol & Wohl, 2010; Heartsill‐Scalley & Aide, 2003).

### 4.3 Other factors affecting stream conditions

Although many of the patterns in environmental variables in our stream are likely to be directly and causatively linked with forest quality at the riparian and catchment‐scale, it is important to recognise that some patterns might be correlative and simply the result of human choices about which areas to develop. For example, in Sabah and other areas of the tropics, logging and development is limited to the lowlands by feasibility and regulations; slopes above 25° are considered unworkable and are generally not released for logging, apart from by helicopter logging (Reynolds et al., 2011), and inaccessible areas are generally avoided. This may mean that catchments and streams selected for oil palm cultivation may already have a suite of characteristics that are different from those that remain forested, rather than differences caused by the clearance itself. High levels of bedrock in higher quality forest catchments may be an example of this, as rocky areas may be less likely to be chosen for oil palm development. However, given that these policies are so widely followed, it may be that some features are still generalizable to high‐quality forest streams, although caused by human selection of which sites to log and convert to oil palm rather than any hydrological or ecological process brought about by forest quality.

### 4.4 Consequences for freshwater ecosystems

Our findings of elevated light levels, temperatures, sand, and nitrate‐N found in disturbed streams, along with lower levels of habitat heterogeneity in terms of leaf and woody matter, rockiness, channel width, and depth, are likely to have substantial impacts on stream ecosystems and the services that streams provide. Temperature increases caused by habitat conversion, particularly in combination with rising temperatures predicted with climate change, are likely to have substantial impacts on freshwater biodiversity and ecosystem functions (Boyero et al., 2011; Hogg & Williams, 1996). Tropical insects in particular have been shown to be vulnerable because they are sensitive to temperature change and are currently living near their optimal temperature (Deutsch et al., 2008). Increases in light levels, nitrate‐N, and decreases in leaf inputs could contribute to a shift to a community dominated by algal growth (Benstead & Pringle, 2004; England & Rosemond, 2004) and substantial changes in stream food webs (Boyero et al., 2011; Covich, Palmer, & Crowl, 1999; Yule et al., 2009). Decreases in channel width, depth, rockiness, and occurrence of dead wood, along with increases in levels of sand in disturbed streams, are likely to reduce habitat complexity and suitable habitat for many benthic invertebrates (Burdon, McIntosh, & Harding, 2013). Simplified benthic habitats are also less able to trap and retain leaf litter, therefore reducing levels of terrestrial organic matter further. These changes in environmental conditions and biota could substantially reduce water clarity, quality, and fish production, with adverse consequences for local people. Furthermore, lower channel width, depth, and high sedimentation in disturbed streams could contribute to increased downstream flood risk.

### 4.5 Management implications

Reduced impact logging has been suggested as a method to decrease damage to remaining forests and soil during timber extraction, through approaches such as skid trail planning, cutting lianas, using culverts in waterways, positioning roads along ridges, and avoiding logging on slopes over 25° (Pinard & Putz, 1996; Putz & Pinard, 1993; Putz et al., 2008; Walsh et al., 2011), all of which could help to minimise negative impacts of logging on freshwaters. Our results indicate that environmental conditions in logged forest streams were often different from old‐growth sites, suggesting that two rounds of conventional selective logging over 10 years earlier were still affecting stream conditions. Although recovery to prelogging levels of water quality has been reported after just a few years in some cases, and for some conditions (e.g., Malmer & Grip, 1994), several other studies found that it took up to 20 years to return to predisturbance levels following logging (Bruijnzeel, 2004; Douglas et al., 1999; Iwata, Nakano, & Inoue, 2003). A study in Sabah found that although erosion rates were substantially lower 21 years after selective logging than they had been during and in a secondary peak 6–10 years after logging, they had not fully returned to normal (Walsh et al., 2011). Our data do not allow for the effects of reduced impact logging compared to conventional logging on freshwaters to be explicitly tested, and no other studies have yet done this. However, given the legacy of logging impacts we have shown, it seems likely that practices that reduce the initial impact of logging on remaining forest would benefit freshwaters.

Retaining forested riparian buffer strips, maintaining headwater and steep‐slope forest cover, and protecting forest patches within catchments have been proposed as ways to help maintain freshwater ecosystems and the services they provide after land conversion (RSPO, 2013). Legislation in Sabah currently stipulates that 20‐m buffers should be maintained on all streams over 3‐m wide (Environment Protection Department (EPD), 2011; Sabah Water Resources Enactment, 1998). Roundtable on Sustainable Palm Oil guidelines also state that in addition to buffer strips (minimum 5‐m wide), there should not be forest clearing or oil palm planting on steep slopes and that soil conservation methods, such as terracing, should be used on 9–25° slopes (RSPO, 2013). Our results indicate that forest quality at both the riparian and catchment‐scale have significant impacts on stream environmental conditions and that the ability of riparian buffer strips to maintain forest‐like stream conditions in oil palm streams depends on the environmental measure being considered. This shows that riparian buffer protection is highly advantageous but apparently not sufficient to maintain stream ecosystems and services fully and highlights the importance of broader scale conservation strategies, such as protection of forest fragments and terracing on steep slopes, being promoted by organisations such as the Roundtable on Sustainable Palm Oil. Other studies also suggest that maintaining catchment‐scale forest cover in addition to the maintenance of riparian buffer strips is important for determining stream conditions (e.g., Allan, 2004; Allan, Erickson, & Fay, 1997; Death & Collier, 2009; Heartsill‐Scalley & Aide, 2003; Sponseller, Benfield, & Valett, 2001; Suga & Tanaka, 2012) and that forest structure and quality have an effect as well as area of forest cover (de Souza et al., 2013). It has also been shown that riparian buffers that have gaps are not enough to offer protection to freshwater ecosystems (Wahl, Neils, & Hooper, 2013). Thus, it seems that catchment‐scale planning and careful protection of designated buffer areas are needed for efforts to be effective.

## 5 CONCLUSIONS

We show that rainforest logging and oil palm agriculture affect a wide range of stream environmental conditions, that both riparian and catchment‐scale forest quality are important in moderating these impacts, and that different stream conditions are affected by disturbance at different scales. Our study also shows that impacts of selective logging upon stream limnology can still be evident over 10 years after habitat disturbance. We consider that maintenance of riparian buffer strips is essential for retaining some forest‐like conditions in streams including aspects of structure, water quality, and organic inputs, but our data suggest that this alone is unlikely to be sufficient to maintain fully forest‐like conditions. We suggest that any logging in the riparian zone should be prevented and that riparian buffer strips alongside streams should be strictly protected. In areas where there is development in the wider catchment, reduced‐impact logging protocols should be used along with added catchment‐scale protection of forest fragments to help maintain freshwater ecosystems and the services that they provide.

## CONFLICT OF INTEREST

We have no conflicts of interest to declare.

## Supporting information


**Figure S1**. Relationship between catchment forest quality PC1 scores and riparian forest quality PC1 scores for each of the sixteen stream sites
**Table S1**. Details of how streams will be affected by proposed future logging at the SAFE Project
**Table S2.** Loading scores showing how original forest quality variables correspond to the principal component summary variables (Catchment and Riparian PC1, PC2, and PC3) produced by PCA, along with the variance in the original variables that is summarised by each component (note that all riparian loadings are multiplied by −1 to make them more readily interpretable)
