# Supplementary material for: The effects of catchment and riparian forest quality on stream environmental conditions across a tropical rainforest and oil palm landscape in Malaysian Borneo
Source: Ecohydrology. 2017 Mar 21;10(4):e1827. doi: 10.1002/eco.1827 (PMC5485068; doi:10.1002/eco.1827)
Supplement: Supplementary file 1 — Figure S1. Relationship between catchment forest quality PC1 scores and riparian forest quality PC1 scores for each of the sixteen stream sites Table S1. Details of how streams will be affected by proposed future logging at the SAFE Project Table S2. Loading scores showing how original forest quality variables correspond to the principal component summary variables (Catchment and Riparian PC1, PC2, and PC3) produced by PCA, along with the variance in the original variables that is summarised by each component (note that all riparian loadings are multiplied by −1 to make them more readily interpretable) [file ECO-10-na-s001.docx]

# Supplementary Materials


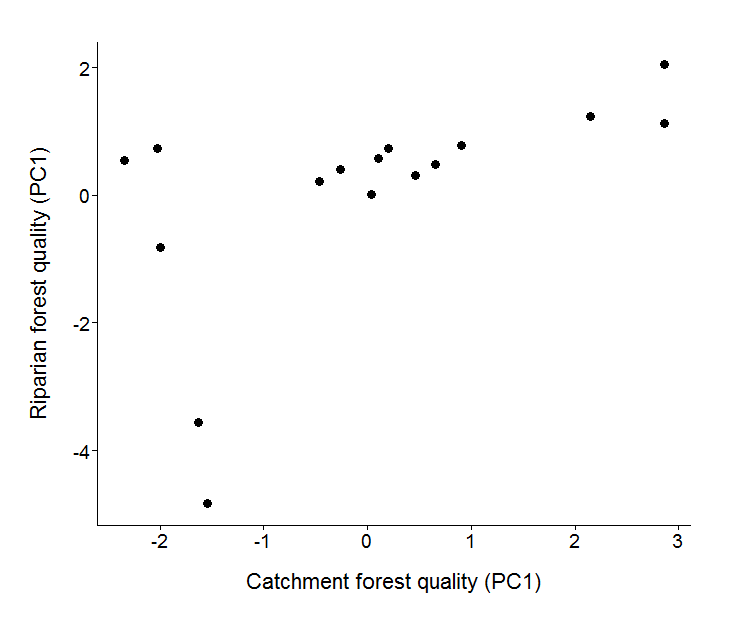


**Figure S1**- Relationship between catchment forest quality PC1 scores and riparian forest quality PC1 scores for each of the sixteen stream sites

**Table S1**- Details of how streams will be affected by proposed future logging at the SAFE Project

| **Stream name in this study** | **Catchment habitat type during this study** | **Catchment habitat type after proposed future logging at the SAFE Project** |
| --- | --- | --- |
| OG-Maliau | Old growth forest, very lightly logged once for building field centre | No change |
| OG-West | Old growth forest | No change |
| OG-Rhinopool | Old growth forest | No change |
| OG-VJR | Old growth forest, some illegal logging | No change |
| LF-1 | Continuous logged forest, selectively logged during the 1970s (removing approximately 113 m^3^ of timber per hectare), and logged again late 1990s-2000s (removing a further 66 m^3^ ha^-1^). Forest today is very heterogeneous with patches of forest with closed canopy interspersed with early re-growth, gaps and roads. | “The 0m stream”  0m buffer left on each side (i.e. no riparian buffer) of the stream, and the remainder of the catchment cleared and planted with young oil palm |
| LF-2 |  | “The 5m stream/new 30m stream”  30m buffer left on each side, catchment cleared and planted with oil palm |
| LF-3 |  | “The 15m stream”  15m buffer left on each side, catchment cleared and planted with oil palm |
| LF-4 |  | “The 30m stream”  30m buffer left on each side, catchment cleared and planted with oil palm |
| LF-5 |  | “The 60m stream”  60m buffer left on each side, catchment cleared and planted with oil palm |
| LF-6 |  | “The 120m stream”  120m buffer left on each side, catchment cleared and planted with oil palm |
| LF-7 | Continuous logged forest, selectively logged during the 1970s (removing approx. 113 m^3^ of timber per hectare), and logged again late 1990s-2000s (removing a further 37 m^3^ ha^-1^ - therefore a little less than at the other stream sites) | “LFE – logged forest edge – stream”  Catchment and survey area remains forested, but oil palm downstream |
| OPB-Gaharu | Oil palm, planted 2009, with a large (mean ~331m, minimum ~75m) forested riparian buffer on each side of the stream | No change |
| OPB-Keruing | Oil palm, planted 2008, with a medium (mean ~68m, minimum ~33m ) forested riparian buffer on each side of the stream | No change |
| OPB-Merbau | Oil palm, planted 2007, with a small (mean ~26m, minimum ~2m ) forested riparian buffer on each side of the stream | No change |
| OP-Selangan Batu | Oil palm, planted 1999, with no forested riparian buffer | No change |
| OP-Binuang | Oil palm, planted 2007, with no forested riparian buffer | No change |

**Table S2-** Loading scores showing how original forest quality variables correspond to the principal component summary variables (Catchment and Riparian PC1, PC2, and PC3) produced by PCA, along with the variance in the original variables that is summarised by each component (note that all riparian loadings are multiplied by -1 to make them more readily interpretable).

| **Measured variables** | **Principal components** | | |
| --- | --- | --- | --- |
| **Riparian forest quality variables** | **Riparian PC1** | **Riparian PC2** | **Riparian PC3** |
| Percentage cover vines | 0.3970 | -0.8339 | 0.3288 |
| SAFE forest quality score | 0.5538 | 0.1802 | 0.2517 |
| Relascope tree density | 0.5014 | 0.5188 | 0.3536 |
| Canopy cover | 0.5332 | -0.0541 | 0.8388 |
| Standard deviation | 1.7620 | 0.8412 | 0.4050 |
| Proportion of variance | 0.7760 | 0.1769 | 0.0410 |
| Cumulative proportion of variance | 0.7760 | 0.9529 | 0.9939 |
|  |  |  |  |
| **Catchment forest quality variables** | **Catchment PC1** | **Catchment PC2** | **Catchment PC3** |
| Above ground biomass (AGB) | 0.5527 | 0.8228 | -0.1327 |
| Forest cover (FCover) | 0.5951 | -0.2781 | 0.7540 |
| Leaf area index (LAI) | 0.5835 | -0.4957 | -0.6433 |
| Standard deviation | 1.6618 | 0.4806 | 0.0864 |
| Proportion of variance | 0.9205 | 0.0770 | 0.0025 |
| Cumulative proportion of variance | 0.9205 | 0.9975 | 1.0000 |
